# Supplementary material for: From sequence to enzyme mechanism using multi-label machine learning
Source: BMC Bioinformatics. 2014 May 19;15:150. doi: 10.1186/1471-2105-15-150 (PMC4229970; doi:10.1186/1471-2105-15-150)
Supplement: Additional file 2 — Java code of ml2db. Additional file ml2db_code.tar.gz contains the Java source code to run the multi-label machine learning experiments and save the results to database. The code’s Javadoc is included. [file 1471-2105-15-150-S2.zip › additional file 2/ml2db/ml2db/doc/main/ml2db/mulan/mulanarff/NeighboursPrinter.html]

NeighboursPrinter


JavaScript is disabled on your browser.


- Overview
- Package
- Class
- Use
- Tree
- Deprecated
- Index
- Help

- Prev Class
- Next Class

- Frames
- No Frames

- All Classes

- Summary:
- Nested |
- Field |
- Constr |
- Method

- Detail:
- Field |
- Constr |
- Method


main.ml2db.mulan.mulanarff

## Class NeighboursPrinter

- java.lang.Object
- - main.ml2db.mulan.mulanarff.NeighboursPrinter

- ---

    

  ```
  public class NeighboursPrinter
  extends java.lang.Object
  ```

  Class to store the neighbours of an instance and print them in a
  human/graphviz readable format. A list of desired instance identifiers can be
  given, if it is undesirable to print the neighbours for all the instances in
  the dataset.

  Version:
  :   5 Oct 2012

  Author:
  :   Luna De Ferrari ldf(at)st-andrews.ac.uk or
      luna.deferrari@st-andrews.ac.uk

- - ### Field Summary

    Fields

    | Modifier and Type | Field and Description |
    | --- | --- |
    | `static java.lang.String` | `ARROW_STYLE` |
    | `static java.lang.String` | `DIRECTED_ARROW` |
    | `static java.lang.String` | `GRAPHVIZ_HEADER` |
    | `static int` | `NUMBER_OF_NEIGHBOURS_1` |
    | `static java.lang.String` | `SHAPE_GREEN_BOX` |
    | `static java.lang.String` | `SHAPE_ORANGE_BOX` |
    | `static java.lang.String` | `UNDIRECTED_ARROW` |
  - ### Constructor Summary

    Constructors

    Constructor and Description

    `NeighboursPrinter(IdMultiLabelInstances unlabelledInstances,
    java.util.Set<java.lang.String> desired)`
  - ### Method Summary

    Methods

    | Modifier and Type | Method and Description |
    | --- | --- |
    | `java.util.Set<java.lang.String>` | `getDesired()` |
    | `uk.ac.standrews.utils.main.maputils.OneToManyMap<IdInstance,IdInstance>` | `getInstanceAndNeighboursMap()` |
    | `IdMultiLabelInstances` | `getInstances()` |
    | `LinearNNSearchSkipInstanceItself` | `getNeighboursSearcher()` |
    | `static void` | `main(java.lang.String[] args)` |
    | `java.lang.String` | `neighboursToString()` |
    | `java.lang.String` | `toUndirectedGraphvizString()` |

    - ### Methods inherited from class java.lang.Object

      `equals, getClass, hashCode, notify, notifyAll, toString, wait, wait, wait`

- - ### Field Detail


    - #### NUMBER\_OF\_NEIGHBOURS\_1

      ```
      public static final int NUMBER_OF_NEIGHBOURS_1
      ```

      See Also:
      :   Constant Field Values


    - #### DIRECTED\_ARROW

      ```
      public static final java.lang.String DIRECTED_ARROW
      ```

      See Also:
      :   Constant Field Values


    - #### UNDIRECTED\_ARROW

      ```
      public static final java.lang.String UNDIRECTED_ARROW
      ```

      See Also:
      :   Constant Field Values


    - #### SHAPE\_GREEN\_BOX

      ```
      public static final java.lang.String SHAPE_GREEN_BOX
      ```

      See Also:
      :   Constant Field Values


    - #### SHAPE\_ORANGE\_BOX

      ```
      public static final java.lang.String SHAPE_ORANGE_BOX
      ```

      See Also:
      :   Constant Field Values


    - #### GRAPHVIZ\_HEADER

      ```
      public static final java.lang.String GRAPHVIZ_HEADER
      ```

      See Also:
      :   Constant Field Values


    - #### ARROW\_STYLE

      ```
      public static final java.lang.String ARROW_STYLE
      ```

      See Also:
      :   Constant Field Values
  - ### Constructor Detail


    - #### NeighboursPrinter

      ```
      public NeighboursPrinter(IdMultiLabelInstances unlabelledInstances,
                       java.util.Set<java.lang.String> desired)
      ```

      Parameters:
      :   `unlabelledInstances` -
      :   `desired` - a list of identifiers of the instances for which the
          neighbours are sought.
  - ### Method Detail


    - #### getDesired

      ```
      public java.util.Set<java.lang.String> getDesired()
      ```


    - #### getInstanceAndNeighboursMap

      ```
      public uk.ac.standrews.utils.main.maputils.OneToManyMap<IdInstance,IdInstance> getInstanceAndNeighboursMap()
      ```

      Returns:
      :   m\_instanceAndNeighbours


    - #### getInstances

      ```
      public IdMultiLabelInstances getInstances()
      ```

      Returns:


    - #### getNeighboursSearcher

      ```
      public LinearNNSearchSkipInstanceItself getNeighboursSearcher()
      ```

      Returns:


    - #### neighboursToString

      ```
      public java.lang.String neighboursToString()
      ```


    - #### toUndirectedGraphvizString

      ```
      public java.lang.String toUndirectedGraphvizString()
      ```


    - #### main

      ```
      public static void main(java.lang.String[] args)
      ```


- Overview
- Package
- Class
- Use
- Tree
- Deprecated
- Index
- Help

- Prev Class
- Next Class

- Frames
- No Frames

- All Classes

- Summary:
- Nested |
- Field |
- Constr |
- Method

- Detail:
- Field |
- Constr |
- Method
